# Comparison of skin microbiota profiles in chronic scratch lesions using tape strip and swab sampling

**DOI:** 10.1016/j.jdin.2026.04.014

**Published:** 2026-04-28

**Authors:** Hye Lim Keum, Woo Jun Sul, Hei Sung Kim

**Affiliations:** a Systems Microbial Ecology Laboratory, Department of Systems Biotechnology, Chung-Ang University, Anseong, Korea; b Department of Dermatology, Incheon St. Mary’s Hospital, The Catholic University of Korea, Seoul, Korea

**Keywords:** chronic scratch lesions, comparison, skin microbiome, swab sampling, tape stripping

*To the Editor:* Chronic scratch lesions, including prurigo nodularis and lichen simplex chronicus, are characterized by epidermal barrier disruption and microbial dysbiosis that may perpetuate the itch-scratch cycle. Swab sampling is widely used in skin microbiome research but primarily captures microorganisms residing on the immediate surface. Tape stripping, in contrast, samples microbes embedded within the superficial stratum corneum, interrogating a biologically distinct epidermal niche.^1^ Because chronic scratch lesions are defined by barrier alteration, sampling depth may influence microbial detection. However, direct within-subject comparisons of these 2 minimally invasive approaches in chronic pruritic dermatoses are limited. To our knowledge, this study provides the first paired comparison of tape strip and swab sampling in lesional and nonlesional skin from the same individuals.

We analyzed paired swab and tape strip samples from 9 patients with chronic scratch lesions (Fig 1). For each subject, lesional skin and contralateral nonlesional control skin were sampled using both methods. Swab samples underwent full-length 16S rRNA sequencing and were analyzed using a previously published pipeline,^2^ whereas tape strip samples were sequenced targeting the 16S rRNA V3 to V4 region. Because amplification regions and bioinformatic workflows differed, analyses were conducted separately for each method, and cross-method comparisons were restricted to community-level ecological metrics rather than direct taxonomic equivalence.^3^

**Fig 1:**
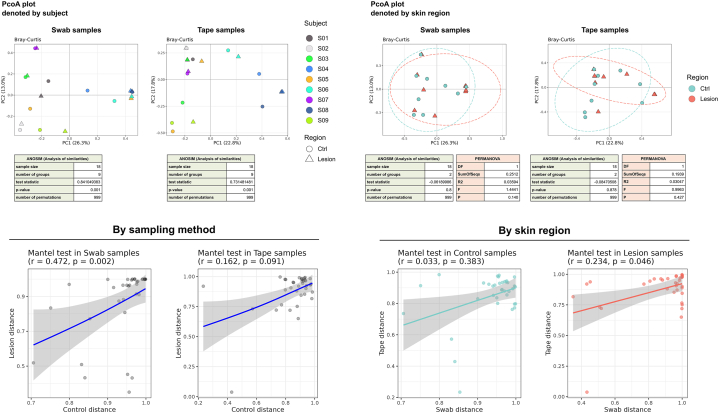
The combination of beta diversity ordination (PCoA) and Mantel test results illustrates how skin microbiome patterns vary by individual, skin region, and sampling method. In the PCoA plots, samples cluster primarily by subject rather than by lesion status, indicating that individual identity is the dominant driver of microbial community structure for both swab and tape strip samples. The Mantel analyses complement this visualization by quantifying similarity patterns: swab samples show preserved subject-specific similarity between lesional and control skin, whereas tape strip samples show greater regional variability. Importantly, swab and tape strip samples are significantly correlated in lesional skin but not in control skin, indicating that lesion-associated microbial signatures are strong and consistently detected across sampling methods, while normal skin microbiota are more sensitive to methodological differences.

Beta diversity analyses based on Bray-Curtis dissimilarity demonstrated strong subject-specific clustering for both methods, indicating that individual identity was the dominant determinant of microbial community structure.^4^ After accounting for subject effects, lesional and nonlesional samples did not form distinct clusters for either method, suggesting that lesion-associated shifts occur within the context of each individual’s baseline microbiome rather than over-riding subject-specific signatures. Alpha diversity did not differ between lesional and control skin for either sampling approach.

Mantel testing revealed method-specific and region-specific concordance patterns. Within swab samples, microbial community distances between lesional and nonlesional skin were significantly correlated, indicating preservation of subject-specific microbial architecture across sites. This correlation was not observed within tape strip samples, suggesting greater sensitivity to local epidermal microenvironmental variation. Notably, microbial community distances between swab and tape strip samples were significantly correlated in lesional skin but not in nonlesional skin. This finding suggests that lesion-associated dysbiosis generates robust ecological signals detectable across sampling depths, whereas the relatively stable and lower-biomass microbiota of normal skin are more susceptible to methodological variability.

Taxonomic profiling revealed broadly similar dominant genera across methods, including *Staphylococcus* and *Cutibacterium.* Tape strip samples showed relatively lower *Cutibacterium* and higher *Corynebacterium* abundance compared with swabs, differences consistent with known effects of sampling depth, biomass, and amplicon target region rather than clear biological discordance.

In conclusion, this within-subject comparison demonstrates that tape stripping captures core ecological patterns of chronic scratch lesions that are concordant with established swab-based profiling, particularly in lesional skin. These findings validate tape stripping as a methodologically sound and biologically complementary approach for microbiome studies in barrier-disrupted dermatoses without implying equivalence at fine taxonomic resolution.

## Conflicts of interest

None disclosed.
